# Therapeutic effects of crocin in alleviating diabetic neuropathy: a preliminary randomized triple-blind placebo-controlled trial

**DOI:** 10.1186/s40780-025-00484-9

**Published:** 2025-08-14

**Authors:** Adeleh Sahebnasagh, Mohammad Ali Feizi, Saeed Hossein Khalilzadeh, Elahe Taghvaei, Fatemeh Saghafi

**Affiliations:** 1 Department of Internal Medicine, Clinical Research Center, Faculty of Medicine, North Khorasan University of Medical Sciences, Bojnurd, Iran; 2 Pharmaceutical Sciences Research Center, School of Pharmacy, Student Research Committee, Shahid Sadoughi University of Medical Sciences and Health Services, Yazd, Iran; 3 Diabetes Research Center, Shahid Sadoughi University of Medical Sciences, Yazd, Iran; 4 Department of Neurology, School of Medicine, Shahid Sadoughi University of Medical Sciences and Health Services, Yazd, Iran; 5 Department of Clinical Pharmacy, Faculty of Pharmacy and Pharmaceutical Sciences Research Center, Shahid Sadoughi University of Medical Sciences and Health Services, Yazd, Iran

**Keywords:** Diabetes mellitus, Type 2, Diabetic neuropathies, Diabetes complications, Crocin, Clinical trial

## Abstract

**Introduction:**

: Diabetic neuropathy is a prevalent and debilitating complication of type 2 diabetes, resulting in functional impairments. Crocin, a bioactive constituent of saffron (*Crocus sativus*), has long been utilized in traditional medicine for its potential therapeutic effects. This study aimed to evaluate the efficacy of Crocin in alleviating neuropathic symptoms in patients with type 2 diabetes.

**Methods:**

In this triple-blind, randomized, placebo-controlled clinical trial, patients with type 2 diabetes and confirmed peripheral neuropathy were randomly assigned to receive either 15 mg/day of Crocin or a matching placebo for 12 weeks. Neuropathic symptoms were assessed using the Total Symptom Score (TSS), Visual Analog Scale (VAS), and the Michigan Neuropathy Screening Instrument (MNSI) at baseline and at 3-week intervals.

**Results:**

Forty-two patients completed the study, with 21 participants in each group. By week 9, the Crocin group exhibited a significantly lower mean TSS (5.1 ± 1.39) compared to the placebo group (6.6 ± 1.00, *P* = 0.005). Similarly, VAS scores, reflecting pain intensity, were significantly reduced in the Crocin group at both weeks 6 and 9 (*P* = 0.019). MNSI scores at week 9 also favored the Crocin group (5.7 ± 1.1 vs. 6.8 ± 0.9, *P* = 0.03).

**Conclusion:**

Crocin may offer promising therapeutic benefits in reducing pain and neuropathic symptoms in patients with type 2 diabetes. Its neuroprotective, antioxidant, and antihyperglycemic properties may contribute to these effects. While the findings support the potential beneficial effect of crocin in the management of diabetic neuropathy, further large-scale and more robust clinical trials are warranted to validate these results.

**Trial registration:**

https//irct.ir/ IRCT20190810044500N8, Registration date 01/01/2021.

## Introduction

Diabetes mellitus and its related complications represent a major global health burden, affecting a considerable portion of the world’s population, with an estimated prevalence of 9.3% in 2023 [1]. One of the most common and disabling complications of diabetes is peripheral neuropathy [2], which develops in approximately 50% of patients during the course of the disease. Among these individuals, nearly half report painful symptoms [3–5]. The clinical manifestations of diabetic peripheral neuropathy (DPN) vary depending on the extent and location of nerve damage and inflammation, including burning and tingling [6], numbness, pain, or muscle weakness, particularly in the extremities. These symptoms can lead to serious functional impairments, such as the difficulty in standing, walking, or grasping objects for prolonged periods [7].

Several risk factors have been implicated in the development and progression of DPN [8], including glucose intolerance, advanced age, longer duration of diabetes, alcohol consumption, and smoking [9]. Currently, pharmacologic options for DPN remain limited. In the United States, only three agents are approved for the treatment of painful DPN: (1) Duloxetine, a serotonin-norepinephrine reuptake inhibitor, (2) pregabalin, an antiepileptic drug, and (3) Tapentadol, a dual-acting opioid receptor agonist and norepinephrine reuptake inhibitor.

Duloxetine and pregabalin are often used as first-line therapies [10]. Pregabalin was the first antiepileptic agent approved by the U.S. Food and Drug Administration (FDA) for the treatment of neuralgia and neuropathic pain including DPN [11]. Clinical trials have demonstrated its efficacy, with numbers needed to treat (NNT) of 6.3 [12]. Meta-analysis of randomized, double-blind placebo-controlled studies in patients with DPN have shown that duloxetine is also effective in reducing the intensity of pain, with outcomes comparable to those of pregabalin and gabapentin [13]. However, duloxetine is associated with adverse effects such as nausea, dizziness, and somnolence, and its NNT varies from 1.3 to 5.1 across studies [14].

Opioid agonists, including tramadol and tapentadol, are considered the second or third-line treatments due to concerns about safety and dependency [15]. While a multicenter, randomized, placebo-controlled study has demonstrated the efficacy of tramadol in improving physical and social functions in patients with DPN, its side effect profile limits its clinical use, including nausea, constipation, headache, and drowsiness [16]. Despite the numerous treatments, managing neuropathic pain remains a significant clinical challenge [17]. The economic impact is also substantial, with the annual cost of DPN in the United States estimated between 4.6 and 13.7 billion, accounting for up to 27% of the direct medical costs related to diabetes [18].

Crocin, a natural carotenoid extracted from Crocus sativus (saffron), has demonstrated multiple pharmacological properties, including antioxidant, anti-inflammatory, anti-cancer, neuroprotective, anti-hypertensive, and cardioprotective properties [19, 20]. Moreover, preclinical studies suggest that Crocin can reduce blood glucose levels and improve insulin resistance [21]. In animal models of neuropathy, chronic administration of Crocin has been shown to attenuate neuropathic pain behaviors, indicating its potential as a novel therapeutic agent in humans [22].

Given the high prevalence of DPN, and the limited efficacy and tolerability of current pharmacologic therapies, this study aimed to investigate the therapeutic effects of Crocin in alleviating diabetic neuropathy in patients with type 2 diabetes.

## Methods

### Patients

Between February and September 2021, a total of 176 patients aged 18 to 85 years with type 2 diabetes mellitus, receiving either oral or parenteral hypoglycemic medications were recruited from the Research Center and affiliated outpatient clinics. Participants who had a TSS ≥ 3 and MNSI ≥ 7.5, with glycosylated hemoglobin A1c (HbA1c) levels below 12% were considered eligible.

Exclusion criteria included the presence of severe medical conditions, limiting the ability to attend follow-up visits, renal dysfunction (creatinine level > 1.7), mild hepatic enzyme elevation (up to five times the upper limit of normal), immunocompromised status, non-diabetic causes of peripheral neuropathy, pregnancy, or unwillingness to use effective contraception during the trial period.

### Assessment

Eligible participants were randomly assigned in a 1:1 ratio to receive either Crocin 15 mg/day or a matching placebo for 12 weeks, using a computer-generated random number table. Crocin and placebo tablets were obtained from Samisaz Pharmaceutical Company.

This triple-blind design ensured that participants, clinical assessors, and data analysts remained blinded to treatment allocation. Additionally, the medical student responsible for administering questionnaires remained unaware of group assignments. Participants continued routine diabetes care, and no alterations were made to their treatment or dietary regimen throughout the study. Concomitant use of any additional therapeutic or complementary interventions was not permitted.

### Assessment tools

Baseline demographic and clinical data, including medical history, current medications, and laboratory data were documented. The primary outcomes included changes in TSS and MNSI scores. Pain intensity was evaluated using the Visual Analog Scale (VAS) as the secondary outcome.

TSS assessed the severity and frequency of four neuropathic symptoms (pain, burning, paresthesia, numbness) based on patient self-report. Symptom severity was graded as absent, mild, moderate, or severe, and frequency as occasional, frequent, or continuous, yielding a total score ranging from 0 (no symptoms) to 14.64 (severe, persistent symptoms) [23]. MNSI included two components: a patient-completed history questionnaire and a clinician-administered physical examination to evaluate sensory neuropathy [24]. VAS measured pain severity on a 10 cm line, with 0 representing no pain and 10 indicating the worst imaginable pain. Assessments were performed at baseline and at 3-week intervals throughout the 12-week trial.

### Ethical considerations

The study was approved by the Ethics Committee of Shahid Sadoughi University of Medical Sciences (Ethics ID: IR.SSU.MEDICINE.REC.1398.130) and conducted in accordance with the declaration of Helsinki. The trial was registered with the Iranian Registry of Clinical Trials (IRCT20190810044500N8). Study participants were provided written informed consent prior to enrollment.

## Sample size

Assuming a two-sided significance level of 5%, a statistical power of 80%, and a standard deviation of 1.7, reported in a similar study, a minimum of 22 participants per group was calculated to detect a mean difference of 2.2 in the MNSI score between the intervention and control groups. Participants randomly allocated to either group using a random number table.

### Statistical analysis

Data analysis was performed using SPSS version 19 software. Quantitative variables were reported as means ± standard deviation, while qualitative variables are expressed as frequency and percentage. Group comparisons were conducted using independent-sample t-test, one-way ANOVA, and repeated-measures test of two-way ANOVA where appropriate. A *P*-value < 0.05 was considered statistically significant.

## Results

**Fig. 1:**
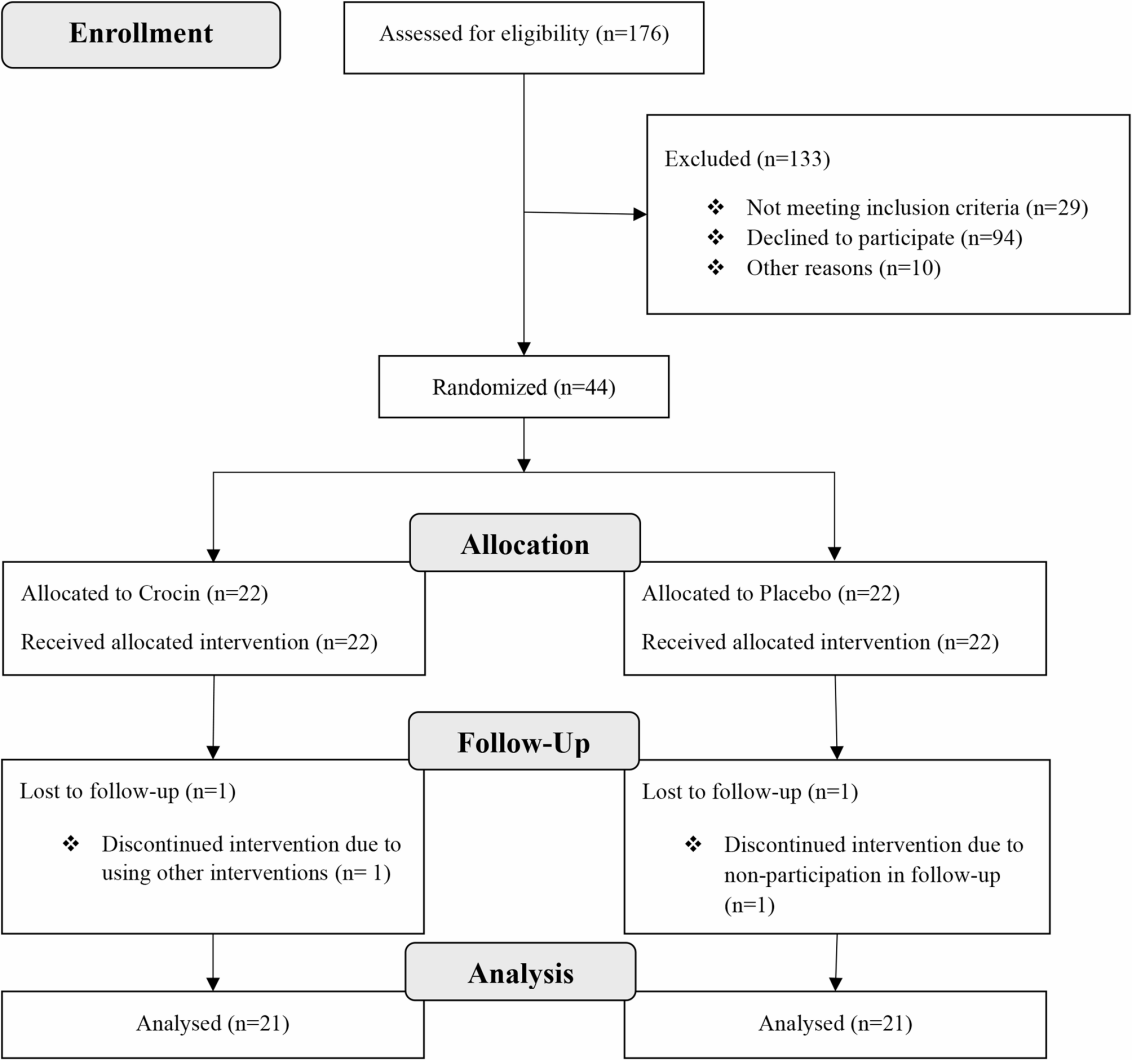
CONSORT flow diagram

The present study was conducted at Yazd Diabetes Clinic in Iran, between February and September 2021. Of the 176 individuals initially assessed for eligibility, 133 were excluded due to not meeting the inclusion criteria or unwillingness to participate .Ultimately, 44 patients with type 2 diabetes and confirmed peripheral neuropathy were enrolled and randomized into two equal groups to receive either Crocin or placebo (22 patients per each group). Two patients (4.5%) prematurely discontinued the study intervention: one in the crocin group due to initiating another intervention and one in the placebo group due to loss to follow-up (Fig. 1). Baseline demographic and clinical characteristics, including age, sex, duration of diabetes, and type of hypoglycemic treatment, were comparable between the two groups (Table 1).

The mean (SD) age of participants was 62.59 (6.3) years, with 16 (38%) female participants, and the average diabetes duration was 17 (6.9) years. Use of insulin and oral or non-insulin injectable medications was evenly distributed at baseline (Table 1).

**Table 1:** Baseline demographic and clinical characteristics of the patients’ population enrolled in the study

Variable	Crocin (*N* = 21)	Placebo (*N* = 21)	*P*-value
Male^1^	15 (71.4)	11 (52.4)	0.089
Age^2^ (year)	62.8 (6.1)	62.38 (6.5)	0.810
BMI^2^ (kg/m^2^)	27.47 (3.5)	26.7 (2.7)	0.452
Diabetes duration^2^ (Years)	18 (7.7)	16 (6.3)	0.361
Laboratory measurements at inclusion^2^			
FBS (mg/dl)	167.43 (45.1)	153.7 (35.21)	0.711
HbA1C (%)	7.1 (1.0)	6.5 (0.9)	0.196
eGFR (mL/min/1.73 m^2^)	53.93 (39.22)	56.01 (19.0)	0.962
Diabetes medications^1^			
Insulin	14 (66.6)	12 (57.1)	0.340
Oral and noninsulin injectable	6 (28.6)	8 (38.1)	0.412

F: Frequency; N: Number; SD: Standard Deviation; BMI: Body Mass Index; kg/m^2^: Kilogram Per Square Meter; FBS: Fasting Blood Glucose; mg/dL: milligrams per deciliter; HbA1C: hemoglobin A1C; eGFR: estimated Glomerular Filtration Rate

1: Data are based on frequency (%)

2: Data are based on mean (SD)

At the end of the 12-week intervention period, the Crocin group demonstrated a significantly lower mean (SD) score on the Total Symptom Score (TSS) index compared to the placebo group [5.13 (1.40) vs. 6.65 (1.28); *P* = 0.005]. This difference became statistically significant only after week 8, with no notable differences observed during earlier weeks. Similarly, the Crocin group reported a significant improvement in the patient-reported MNSI symptom questionnaire score at week 12 compared to placebo (*P* = 0.033). However, no significant differences were detected between groups in MNSI scores derived from physical examination across the study period. Pain intensity, as assessed by VAS scores, was significantly reduced in the Crocin group at both week 8 (*P-value* = 0.048) and week 12 (*P-value* = 0.019), supporting the efficacy of Crocin in alleviating neuropathic pain symptoms (Table 2).

**Table 2:** Summary of primary and secondary outcome results of randomized patients

Outcome	Crocin (*N* = 21)	Placebo (*N* = 21)	*P*-value
	Mean (SD)		
TSS			
Baseline	7.87 (1.41)	8.23 (1.70)	0.934
4th week	7.80 (1.65)	7.82 (1.37)	0.922
8th week	6.12 (1.15)	7.14 (0.84)	0.186
12th week	5.13 (1.40)	6.65 (1.28)	0.005
**VAS**			
Baseline	6.71 (1.10)	6.47 (1.39)	0.901
4th week	6.22 (1.39)	6.34 (1.25)	0.924
8th week	5.18 (1.00)	6.11 (0.84)	0.048
12th week	4.61 (0.93)	5.72 (1.00)	0.019
**MNSI**			
Symptoms stated by the patient			
Baseline	8.14 (1.32)	7.90 (1.54)	0.992
4th week	7.38 (0.78)	7.65 (1.00)	0.831
8th week	6.20 (1.13)	7.22 (0.84)	0.069
12th week	5.76 (1.11)	6.82 (0.91)	0.033

TSS: Total Symptom Score; VAS: Visual Analogue Scale; MNSI: Michigan Neuropathy Screening Instrument; N: Number; SD: Standard Deviation

## Discussion

This study evaluated the therapeutic potential of Crocin in alleviating diabetic peripheral neuropathy. Our findings demonstrated that although there were no significant differences between the Crocin and placebo groups at baseline, notable improvements emerged in the Crocin group during the follow-up period. Specifically, significant reductions in pain severity were observed in the Crocin group, as evidenced by lower VAS scores at weeks 6 and 9. Furthermore, by week 9, the Crocin group exhibited significantly lower TSS and MNSI-Q scores compared to the placebo group. Therefore, Crocin may be effective in reducing diabetic neuropathy pain in patients with DPN after eight weeks of treatment.

Diabetic neuropathy is a debilitating complication of diabetes, affecting sensory, motor, and autonomic nerves. It typically presents with symptoms such as burning pain, paresthesia, and numbness, particularly in the lower limbs. These symptoms tend to worsen at night, impairing patients’ sleep and quality of life. Effective management of DPN remains a clinical challenge and is primarily focuses on symptom relief and prevention of further nerve damage [25]. Chronic hyperglycemia is thought to play a key role in the pathogenesis of DPN by inducing mitochondrial dysfunction, promoting production of reactive oxygen species (ROS), impairing neuronal blood flow, reducing the availability of neurotrophic factors, and slowing nerve conduction. Without proper glycemic control, these changes can lead to irreversible damage to the peripheral nervous system.

Natural compounds, including plant-derived substances, have long been investigated for their neuroprotective properties. Crocin, a carotenoid derived from Crocus sativus (saffron), has attracted considerable attention due to its broad pharmacological profile. Previous studies have shown that Crocin possesses antioxidant, anti-inflammatory, anti-apoptotic, and neuroprotective properties. It also appears to improve cognitive function, reduce stress, and protect against DNA damage and neuronal injury induced by oxidative stress or cerebral ischemia [26–30].

Several studies have investigated the effects of saffron and its active ingredient, Crocin, on various types of pain, including neuropathic pain. Our findings are consistent with preclinical studies indicating the efficacy of Crocin in models of neuropathic pain. For instance, in chronic constriction injury (CCI) models, both aqueous saffron extracts and Crocin reduced oxidative stress, inflammation, and neuronal apoptosis [31]. Amin et al. also demonstrated the effectiveness of saffron extracts in alleviating various forms of nerve pain, highlighting their potential as adjuncts to standard pharmacotherapy [32]. Moreover, another study revealed that Crocin, at a dose of 80 mg/kg, mitigated nerve damage and reduced pain responses in a cold allodynia test [33]. These findings are in agreement with our study, indicating that Crocin’s reduction of neuropathic pain after eight weeks may be attributed to its neurodegenerative effects.

Similarly, another investigation reported that saffron and Crocin extract (30 mg/kg) attenuated thermal hyperalgesia and mechanical allodynia by day 26, with these effects sustained through day 40. However, the study also reported that Crocin was ineffective at a lower dose (15 mg/kg), suggesting a dose-dependent response [34]. In our trial, only a single daily dose of 15 mg was administered, and dose-response relationships were not explored. Beyond diabetic neuropathy, Crocin has shown promise in chemotherapy-induced peripheral neuropathy (CIPN). Evidence suggests that Crocin may alleviate CIPN symptoms with fewer side effects compared to traditional agents such as antidepressants, lamotrigine, and gabapentin [35].Several mechanisms may contribute to Crocin’s effectiveness in reducing diabetic neuropathic pain. One proposed mechanism involves the modulation of intracellular calcium levels. Crocin has been shown to inhibit the influx of extracellular Ca^2+^ and release Ca^2+^ from intracellular stores in the endoplasmic reticulum, thereby reducing intracellular calcium concentrations [36]. This calcium reduction may lead to vasodilation and subsequent tissue hyperemia, which could improve peripheral nerve perfusion [37].

Oxidative stress and ROS overproduction are pivotal in the pathogenesis of neuropathic pain. There is substantial evidence supporting the role of antioxidant agents in alleviating neuropathic pain. Both ethanolic and aqueous extracts of saffron, Crocin in particular, exhibit significant antioxidant activity by reducing lipid peroxidation. These compounds inhibit deoxyribose degradation in red blood cell membranes and liver microsomes, thereby preserving cellular integrity. Recent studies have also demonstrated that saffron and Crocin can protect against hippocampal oxidative damage and cognitive dysfunction induced by chronic stress, suggesting that Crocin’s antioxidant and potential glucose-lowering properties may play a central role in alleviating neuropathic pain [38].

Furthermore, Crocin has been shown to reduce acrylamide-induced neurotoxicity in Wistar rats by attenuating oxidative stress. Dose-dependent administration of Crocin suppressed lipid peroxidation and increasing glutathione (GSH) levels, further confirming its neuroprotective effects [39].

In PC-12 cells, Crocin inhibited lipid peroxidation formation, partially restored superoxide dismutase (SOD) activity, preserved neuronal morphology and reduced apoptotic cell death, highlighting its cytoprotective and anti-apoptotic properties [40–42].

Another proposed mechanism by which Crocin reduces diabetic neuropathy pain is its involvement in glycemic control and glucose metabolism [33, 43]. A study by Kianbakht et al. demonstrated that saffron increased blood insulin levels without causing liver or kidney toxicity in diabetic rats, implicating Crocin, Croscetin, and Safranal in this effect [44]. Additional studies have shown that Crocin reduces blood glucose levels and increases the expression of phosphoenolpyruvate carboxykinase 1 (PCK1), a key enzyme involved in gluconeogenesis and glucose homeostasis [45]. Moreover, the neuroprotective effects of Crocin and Safranal may therefore be partially mediated through their anti-hyperglycemic and antioxidant properties [46]. While our study did not evaluate blood glucose levels, these findings align with the observed reduction in neuropathic symptoms.

### Study limitations

While this clinical trial yielded promising findings, the limitations of the study should be acknowledged when interpreting the results. First, the sample size was relatively small. Although 176 individuals were assessed for eligibility, only 42 patients ultimately completed the study, which may affect the generalizability of the findings. Second, the study was limited to 12 weeks, potentially insufficient to assess the long-term efficacy and safety of Crocin in managing diabetic neuropathy. Additionally, the primary outcome measures included patient-reported instruments such as the MNSI, which, despite being validated tool, may be subject to response bias and variability in individual perception of symptoms. The use of a single fixed dose (15 mg/day), chosen based on previous studies, represents another limitation. The lack of dose comparison restricts our understanding of the optimal therapeutic dose or potential dose-response effects. While nerve conduction and use of objective diagnostic tools are ideal for assessing diabetic neuropathy, since the present study was a preliminary trial, our focus was on evaluating patient-reported outcomes using validated clinical scales (TSS, MNSI, and VAS).

It should be noted that although the mean reduction in symptom score was statistically significant, the absolute change may be considered modest in terms of clinical practice and the clinical relevance of these changes should be interpreted with caution. Therefore, further trials incorporating objective neurological assessments and validated clinical benchmarks are warranted to confirm whether these changes translate into meaningful functional or quality-of-life improvements for patients with diabetic neuropathy.

Another limitation of our study was not evaluating the specific anatomical sites of peripheral neuropathy, separately. Although the validated instruments applied in this clinical trial primarily reflect symptoms in the lower limbs which are commonly affected in diabetic neuropathy, we did not collect data on the precise localization (e.g., distal versus proximal involvement, or foot versus hand symptoms). To determine treatment effects based on the location of neuropathic pain, future studies with detailed mapping of neuropathic involvement should take this limitation into account.

Future research should include larger sample sizes, extended follow-up durations, exploration of various dosing regimens, and objective electrophysiological assessments. Investigating the precise molecular mechanisms by which Crocin alleviates diabetic neuropathy will provide deeper insights, particularly its role in neuroinflammatory pathways, oxidative stress, and intracellular calcium signaling. Furthermore, studies assessing Crocin in combination with other conventional treatments (e.g., analgesics, anti-inflammatories) may elucidate potential synergistic effects.

## Conclusion

This clinical trial demonstrated that Crocin significantly alleviated neuropathic symptoms in patients with type 2 diabetes mellitus. The observed therapeutic effects may be attributed to Crocin’s antioxidant pro potential glucose-lowering properties, modulation of calcium signaling, and potential glucose-lowering properties. Although the findings are promising, further well-powered studies with extended follow-up and mechanistic evaluations are warranted to confirm Crocin’s efficacy and establish its role as a complementary treatment for diabetic peripheral neuropathy.

## Data Availability

No datasets were generated or analysed during the current study.
